# Does a quality improvement campaign accelerate take-up of new evidence? A ten-state cluster-randomized controlled trial of the Institute for Health Improvement’s Project JOINTS

**DOI:** 10.1186/s13012-017-0579-7

**Published:** 2017-04-17

**Authors:** Eric C. Schneider, Melony E. Sorbero, Ann Haas, M. Susan Ridgely, Dmitry Khodyakov, Claude M. Setodji, Gareth Parry, Susan S. Huang, Deborah S. Yokoe, Don Goldmann

**Affiliations:** 1 RAND Corporation, Santa Monica, CA USA; 2 Division of General Medicine and Primary Care, Brigham and Women’s Hospital, Boston, MA USA; 3 Department of Health Policy and Management, Harvard School of Public Health, Boston, MA USA; 4 Institute for Healthcare Improvement and Department of Pediatrics, Boston Children’s Hospital, Boston, MA USA; 5 Division of Infectious Diseases and Health Policy Research Institute, University of California Irvine School of Medicine, Irvine, CA USA; 6 Division of Infectious Diseases, Brigham and Women’s Hospital and Harvard Medical School, Boston, MA USA; 7 The Commonwealth Fund, One East 75th Street, New York, NY 10021 USA

## Abstract

**Background:**

A decade ago, the Institute for Healthcare Improvement pioneered a quality improvement (QI) campaign, leveraging organizational and personal social networks to disseminate new practices. There have been few rigorous studies of the QI campaign approach.

**Methods:**

Project JOINTS (Joining Organizations IN Tackling SSIs) engaged a network of state-based organizations and professionals in a 6-month QI campaign promoting adherence to three new evidence-based practices known to reduce the risk of infection after joint replacement. We conducted a cluster-randomized trial including ten states (five campaign states and five non-campaign states) with 188 hospitals providing joint replacement to Medicare. We measured adherence to the evidence-based practices before and after the campaign using a survey of surgical staff and a difference-in-difference design with multivariable adjustment to compare adherence to each of the relevant practices and an all-or-none composite measure of the three new practices.

**Results:**

In the campaign states, there were statistically significant increases in adherence to the three new evidence-based practices promoted by the campaign. Compared to the non-campaign states, the relative increase in adherence to the three new practices in the campaign states ranged between 1.9 and 15.9 percentage points, but only one of these changes (pre-operative nasal screening for *Staphylococcus aureus* carriage and decolonization prior to surgery) was statistically significant (*p* < 0.05). On the all-or-none composite measure, adherence to all three evidence-based practices increased from 19.6 to 37.9% in the campaign states, but declined slightly in the comparison states, yielding a relative increase of 23 percentage points (*p* = 0.004). In the non-campaign states, changes in adherence were not statistically significant.

**Conclusions:**

Within 6 months, in a cluster-randomized trial, a multi-state campaign targeting hospitals and professionals involved in surgical care and infection control was associated with an increase in adherence to evidence-based practices that can reduce surgical site infection.

**Electronic supplementary material:**

The online version of this article (doi:10.1186/s13012-017-0579-7) contains supplementary material, which is available to authorized users.

## Background

Improving the quality and safety of care is a key national objective in the United States. The Affordable Care Act of 2010 encourages the use of evidence-based practices and innovative delivery models to drive quality improvement (QI). Several methods, including academic detailing, activation of opinion leaders, and QI collaboratives, have been shown in the past to improve health care quality [1–3]. Nevertheless, most efforts to accelerate the widespread improvement through the use of new evidence-based clinical practices have fallen short of expectations. Improvement projects often take substantial time and organizational resources and may not succeed despite several iterations. The use of academic detailing techniques and mobilization of opinion leaders can be difficult to bring to scale. QI collaboratives may be inefficient for rapidly spreading new practices, and studies of collaboratives have produced mixed results about the components essential for success [3]. These uncertainties may give pause to organizations weighing whether to invest in a collaborative [4].

A decade ago, the Institute for Healthcare Improvement (IHI) pioneered a potentially more efficient and effective model for engaging organizations in making evidence-based changes to practice: the QI campaign. First applied in IHI’s 100,000 Lives Campaign [5] between 2004 and 2006 and the 5 Million Lives Campaign (designed to reduce adverse events in hospitals) [6] between 2006 and 2008, the model used several political campaign techniques to achieve improvement. These included targeted messages and communication strategies, a “whistlestop tour,” “mentor” hospitals, and easily accessible expert faculty and campaign materials (such as practical “How-to Guides”) [7]. To leverage peer and social network effects, the model sought endorsement and co-messaging by relevant professional societies. IHI organized a “Rapid Spread Network” (RSN), a volunteer network of state-level organizations or “nodes” (e.g., state hospital associations and CMS-sponsored quality improvement organizations) to facilitate recruitment of local organizations and professionals and motivate their pursuit of the campaign’s improvement goals [8, 9]. Several communication strategies such as regular calls with the node organizations, an “office hours” function, and expert speakers amplified the improvement messages generated by IHI.

Randomized controlled studies of IHI-style campaigns have not been performed. The available evidence on campaigns is incomplete and somewhat controversial [10]. A comparison of expected versus observed hospital mortality before and after the 100,000 Lives Campaign suggested a reduction of more than 100,000 in-hospital deaths relative to a projected number of deaths, but a formal independent evaluation was not performed. The 5 Million Lives Campaign did not include measures of adverse events, making it difficult to evaluate its effectiveness in reducing harm to hospitalized patients. Other national initiatives that included some features of campaigns (such as the Get With the Guidelines stroke, heart failure, and acute myocardial infarction initiatives) lacked control groups, leaving uncertainty about how much improvement was attributable to these initiatives [11, 12].

In 2011, IHI designed a new campaign to accelerate uptake of evidence-based practices that had been shown to prevent surgical site infections (SSI) associated with hip and knee arthroplasty. Project JOINTS (Joining Organizations IN Tackling SSIs) was a multi-faceted, disciplined initiative that used the Rapid Spread Network (defined above), methods, and tools developed during previous IHI campaigns to influence orthopedic practices and hospitals performing hip and knee arthroplasty [13]. Rather than launch nationally as other campaigns have done, Project JOINTS was introduced in a cluster-randomized fashion to enable a rigorous, independent evaluation of its efficacy in promoting uptake of new evidence-based practices.

## Methods

### Overview

We hypothesized that Project JOINTS would accelerate the take-up of evidence-based infection prevention practices. The network theory predicts both direct and indirect effects of a campaign. Some local hospital leaders and clinicians will participate directly in IHI campaign activities and adopt the new evidence-based practices while others will become aware of the practices indirectly through peers in the same community or state. Because a campaign relies on indirect transmission through social networks, the control group in a randomized control trial of a campaign must be geographically distinct to avoid cross-contamination.

For Project JOINTS, the evaluation team designed a state-level cluster-randomized trial involving five geographically distinct pairs of states selected and matched on characteristics described below. One state from each pair as selected at random to receive the 6-month Project JOINTS campaign (the other state served as a matched control). To measure changes in the relevant evidence-based practices, RAND conducted a survey of staff participating in orthopedic surgery in all ten states before and after Project JOINTS. The project was reviewed and approved by the RAND Institutional Review Board.

### Intervention

A campaign is based on three main pillars: (1) the evidence supporting new clinical practices that improve care; (2) the evidence that bundling such practices can improve uptake; [14] and (3) the theory underlying the actions that make up the campaign. The three new evidence-based practices not consistently applied to orthopedic surgical patients prior to 2010 were as follows:

1. Screen patients for nasal *Staphylococcus aureus* (SA) carriage and decolonize SA carriers with 5 days of intranasal mupirocin and CHG bathing (minimum three consecutive days of daily use) in the days immediately preceding surgery [15, 16]
2. Instruct patients, regardless of SA carriage, to bathe or shower with chlorhexidine gluconate (CHG) for at least 3 days before surgery [17–19]
3. Use an alcohol-containing antiseptic agent for pre-operative skin preparation [20, 21]

Project JOINTS built on the notion of bundling [14]. IHI designed and encouraged implementation of a five-component “enhanced” SSI prevention bundle including the three new evidence-based practices above and two well-established Surgical Care Improvement Program (SCIP) practices (Table 1)*.*

**Table 1:** Five bundle components of Project JOINTS and the corresponding measures of evidence-based practices (in italics)^a^

Bundle component/measures		Evidence/rationale for inclusion
New evidence-based infection control practices		
1. Nasal screening and decolonization		Screen patients for SA carriage and decolonize SA carriers with 5 days of intranasal mupirocin [15, 16] because SA nasal colonization has been shown to be associated with an increased risk for SSI
*1.a.*	*Pre-operative nasal screening for Staphylococcus aureus (SA) carriage*	
*1.b.*	*Intranasal mupirocin prescribed for methicillin-resistant S. aureus (MRSA) carriers*	
*1.c.*	*Intranasal mupirocin prescribed for methicillin-resistant S. aureus (MRSA) carriers*	
*2.*	*Chlorhexidine bathing 3 or more times pre-operatively*	Instruct patients to bathe or shower with chlorhexidine gluconate (CHG) for at least 3 days before surgery [17–19] to reduce bacterial colonization of the skin prior to surgery (regardless of SA carriage)
*3.*	*Alcohol-containing antiseptic used to prepare skin in operating room*	Use an alcohol-containing antiseptic agent for pre-operative skin preparation [20, 21] because the use of alcohol in addition to a long-acting antiseptic agent provides superior protection against SSIs
Previously promoted infection control practices from the Surgical Care Improvement Program (SCIP)		
4. Peri-operative intravenous antibiotics		*SCIP practice 1*
*4.a.*	*Timely receipt of prophylactic intravenous antibiotics*	
*4.b.*	*Peri-operative intravenous vancomycin for MRSA carriers*	
*5.*	*Appropriate hair removal technique (not razor)*	*SCIP practice 2*

^a^For each infection control practices, respondents answered the following question: “Thinking about the patients undergoing hip and knee surgery whose care you are involved in, to the best of your knowledge, *estimate how frequently* each of the following processes occurs for those patients who do not have any contra-indications.” Response options were percentages from 0 to 100% in 10% increments and “do not know”

The campaign’s design reflected the previously described campaign theory [8, 9]. IHI recruited state organizations from the RSN developed during the 100,000 Lives and 5 Million Lives Campaigns. The state organizations reached out to hospitals in their states inviting them to participate in the campaign, disseminating information about the campaign and its evidence-based practices, and assisting IHI in publicizing activities described below and in a prior publication [13].

IHI developed a logic model summarizing how campaign activities were designed to achieve specified goals (Additional file 1). This model was refined in collaboration with the evaluation team to inform its selection of evaluation measures. IHI developed intervention materials (including a “How-to Guide,” evidence reviews, a summary of the “business case” for the interventions to prevent SSIs, and tip sheets for surgeons and other providers, and patients and families), created and maintained a project website and email listserv, and offered a range of learning opportunities (including webinar calls, faculty-led office hours, and town hall meetings). Hospital and practice staff who participated in Project JOINTS (typically quality or safety improvement leaders and staff with oversight of infection prevention) received access to a password-protected IHI website maintained by IHI.

In addition to SSI prevention in hip and knee arthroplasty, the Project JOINTs “How-To Guide” also addressed important elements of the “Model for Improvement,” developed by Associates in Process Improvement [22] and later adapted by IHI for use in its campaigns. These elements included (1) the development of a QI plan (including explicit aims and a measurement framework); (2) small-scale tests of change (“PDSA cycles”) to refine implementation approaches through iterative learning; and (3) reliance on multi-disciplinary implementation teams. To enhance credibility with clinicians, IHI collaborated with relevant professional organizations, recruiting faculty from the American Academy of Orthopedic Surgeons (AAOS) and the American Association of Hip and Knee Surgeons (AAHKS) and obtaining endorsement from the AAHKS.

### Measuring adherence to evidence-based practices used to prevent surgical site infection

To measure adherence to the evidence-based practices used to prevent surgical site infection in the study hospitals, RAND’s evaluation team developed a written survey that could be completed by front-line professionals involved in the direct care of patients (orthopedic surgeons, nurses, and operating room staff); this survey was administered before and after the Project JOINTS campaign. Table 1 describes the approach to the primary study outcomes and their associated survey questions. The questions addressed three actions (or subcomponents) constituting the nasal screening and decolonization process (new practice 1): screening for SA (1.a.); mupirocin nasal decolonization for methicillin-resistant SA (MRSA) (1.b.) and mupirocin nasal decolonization for methicillin-sensitive SA (MSSA) (1.c.). It included a question on skin decolonization using CHG (new practice 2) and a question on alcohol-containing antiseptic in the operating room (new practice 3). Respondents were asked to indicate the frequency of use of each practice among patients undergoing hip or knee arthroplasty. Response options ranged from 0 to 100% in 10% increments and a “do not know” option. The survey separated practices 1 and 2, but these may not be effective if not used together.

The survey also included questions on evidence-based practices previously promoted by an established SSI prevention program (the Surgical Care Improvement Program or SCIP): a question about initiation of peri-operative intravenous antibiotics within the appropriate time interval prior to the start of arthroplasty (4.a.), a question about the use of intravenous vancomycin for prophylaxis for patients known to be MRSA carriers (4.b.), and a question on the use of appropriate hair removal techniques (5). Project JOINTS reinforced the use of these previously promoted practices. In addition, to evaluate partial adherence to new practices, the survey included a question about the use of chlorhexidine bathing one or two times preoperatively rather than the three times recommended by Project JOINTS [23]. The pre- and post-intervention surveys contained a question about the respondent’s awareness of each of the short list of active and recent QI projects, including Project JOINTS.

The baseline survey (but not the follow-up survey) included questions about factors that might affect the responsiveness of an organization to a QI intervention. These questions, adapted from previous patient safety surveys, addressed barriers to changing SSI prevention protocols, allowed the respondent to grade the pre-operative and surgical units for their performance on patient safety generally, and asked the respondent to rate those units on their culture of excellence in QI [24]. Items were included to assess the hospital’s management support for various patient safety activities, the use of temporary staff, and staff turnover. It included questions about respondent characteristics including type of staff position and number of hours worked each week.

### Hospital data and multistage sampling approach

RAND researchers obtained hospital characteristics from the FY 2011 American Hospital Association data including hospital ownership, affiliation with a hospital system/chain, teaching status (member of the Council of Teaching Hospitals), presence of a residency program, rural location, and number of beds. Hip and knee replacement volumes were based on 2009 Medicare Hospital Compare data.

Starting with organizations in 36 states that had participated in previous IHI campaigns, IHI staff identified 18 states that had state-level organization leaders willing to assist with Project JOINTS and willing to be randomized to either beginning Project JOINTS within the year (intervention) or waiting for a year to start Project JOINTS after it was opened to other states (control). From these 18 states, RAND researchers selected ten states that had a minimum of 20 hospitals performing hip and knee arthroplasty and could be sorted into five pairs that were relatively well matched on (1) the number of hospitals performing at least 100 hip and knee arthroplasties for Medicare beneficiaries each year (based on 2009 CMS Compare data), (2) whether the state required reporting of serious adverse events, and (3) whether the state had non-payment policy for serious reportable events. After the five state pairs were identified, one state was selected at random from each pair to initiate Project JOINTS while the other served as a control. IHI staff contacted the state organization leader of the intervention states to confirm the start date of the campaign and the control states to express interest in starting the campaign after the end of the trial.

Based on power calculations, accounting for the number of respondents per hospital, two-stage response rates, the clustering of respondents by hospital, and the correlation between baseline and follow-up surveys, we estimated that a starting sample of approximately 1200 (6 respondents times 200 hospitals) would yield a final sample with 80% power to detect an eight percentage point change from an assumed baseline bundle adherence rate of 40%. RAND researchers selected 20 hospitals at random from among all hospitals in each state that performed a minimum of 100 hip or knee arthroplasties for Medicare beneficiaries. In the two smallest states, all hospitals meeting the inclusion criteria were included. Members of the research team contacted each selected hospital to request a list of surgeons, nurses, and other staff involved in direct care of patients undergoing hip or knee arthroplasty, supplementing these lists with directory searches for orthopedic surgical practices affiliated with each hospital. From each of these hospital lists, six staff members were selected at random for inclusion in the staff survey sample.

### Survey data collection

Beginning in February 2012, RAND’s Survey Research Group sent all sampled staff members both mail and email invitations to complete the baseline version of the survey either by mail or via a website along with an offer of a $50 gift card incentive. Weekly reminders were sent to non-responders by email or postcard, followed by a second copy of the survey and up to ten phone prompts. In October 2012, after the 6-month Project JOINTS intervention, the post-intervention version of the survey was sent to all sampled individuals from hospitals with at least one respondent to the baseline survey using the same follow-up protocol as was used the baseline survey. The individual response rate to the baseline survey was 41.2%, and response rate for the follow-up survey was 51.6%.

### Analysis

We excluded respondents who failed to answer the first item on the survey (“In your staff position, do you typically have direct interaction or contact with patients?”) and those who had missing or “do not know” responses to six or more of the eight questions used as study outcomes. We excluded hospitals that lacked a valid survey response in both the pre-intervention and post-intervention periods. Using survey data, we compared the characteristics of individual respondents in campaign and non-campaign states. Using the 2011 AHA data, 2009 Hospital Compare data, and baseline survey response data listed above, we compared the hospital characteristics in the campaign and the non-campaign states.

To measure the effect of the campaign, we used an intention-to-treat approach, estimating the impact of being located in a campaign state (vs. non-campaign state) using a respondent-level analysis. The dependent variables were pre-specified as the respondent-level reported percentage adherence to each of the evidence-based components of the SSI reduction bundle and an all-or-none composite calculated as the percentage of respondents reporting >90% adherence by the hospital to all three new evidence-based components of the SSI reduction bundle. The stringent all-or-none and >90% adherence criteria were selected to measure whether the campaign drove highly-reliable adoption of the entire package of evidence-based practices.

To account for potential confounding between the study outcomes and residual non-random allocation of respondent and hospital characteristics that can occur despite cluster-randomization, we adjusted for these characteristics at baseline using a hierarchical model. The model included a state and a hospital level random effect for each of the dependent variables, within a doubly robust method for adjustment, calculating and using propensity score weights [25, 26]. The goal was to make the distributions of the respondent characteristics from the non-campaign states resemble the distribution characteristics of the respondents from the campaign states. The hospital mean scores on baseline survey responses for patient safety scales (management support for patient safety, teamwork across units, handoffs and transitions) were also entered into the models.

The propensity score weights were estimated (in the TWANG package in R [27]) using both hospital and respondent characteristics. Hospital characteristics included ownership, affiliation with a hospital system, membership in the counsel of teaching hospitals (COTH), residency program, rural location, number of beds, 2013 Medicare volume of hip and knee replacement surgeries, and the hospital mean values of each of the three patient safety scales from the survey. Individual respondent characteristics included professional position (surgeon; physician assistant (PA)/nurse practitioner (NP); other), whether the staff member had direct contact with patients, the type of surgical involvement (pre-operative contact only; peri-operative contact only; both pre-and peri-operative contact), years in the current specialty or profession, years in current hospital, years in the current unit, hours worked per week (less than 20; 20–39; 40+), and a flag assigned if the individual had missing/do not know responses to three or more of the study outcomes.

We performed a difference-in-difference analysis for each study outcome comparing the change from baseline to post-intervention in campaign states and matched non-campaign states. Each outcome was regressed on the main effects for intervention (1 = intervention state; 0 = control state) and time period (1 = post-intervention, 0 = baseline). An interaction term including both the intervention and time period variables was used to test the effect of the intervention. Accounting for baseline differences allows us to account for unmeasured differences between campaign and non-campaign sites that are constant over time. Each “frequency of use” study outcome (ranging 0 to 100% in 10% increments) was modeled linearly. The dichotomous variable representing the all-or-none composite (at least 90% use of all evidence-based practices) was modeled using a logistic regression. We included matched pair blocks as fixed effects to account for the matching of states. Potential confounders were included as fixed effects. To ease interpretation, we calculated the least-squares means (LS-means) from the models. In this procedure, each model was used to predict the expected value at baseline and follow-up in the intervention and control states with all other fixed effects set to their mean values.

## Results

After exclusions, 165 of the 188 sampled hospitals (88%) had at least one valid survey response in both the baseline and post-intervention time periods. Despite the state-level matched pairs, respondents in the campaign and non-campaign states differed in professional position, the length of time in specialty or profession, and the length of time working within the hospital and unit, but not in the proportions involved in pre-operative and peri-operative care or the number of hours worked in the hospital each week (Table 2). Study hospitals in the campaign and non-campaign states were relatively similar on baseline characteristics including the average number of respondents per hospital, the volume of hip and knee arthroplasties for Medicare beneficiaries, ownership, affiliation with hospital networks, urban vs. rural location, teaching status, and number of beds (Table 3). Hospitals were similar also on reported measures of quality and safety culture including management support for patient safety, teamwork across units, and transitions and handoffs.

**Table 2:** Characteristics of survey respondents at baseline (*n* = 549)

	Campaign states	Non-campaign states	
	*n* = 235	*n* = 314	
	*%*		*p* ^a^
Staff position			*<0.001*
Surgeon	73.9	55.8	
Physician assistant/nurse practitioner	13.7	9.6	
Other^b^	12.4	34.6	
Surgical involvement			0.65
Pre-operative and peri-operative	77.7	74.4	
Peri-operative only	19.3	21.8	
Pre-operative only	3.0	3.9	
Years working in current specialty or profession			*0.012*
Less than 6 years	14.1	22.6	
6 to 10 years	16.2	19.4	
More than 10 years	69.7	58.0	
Years working in this hospital			*<* *0.001*
Less than 6 years	23.0	38.8	
6 to 10 years	20.4	20.2	
More than 10 years	56.5	41.0	
Years working in current unit			*0.003*
Less than 6 years	27.8	40.5	
6 to 10 years	20.7	21.4	
More than 10 years	51.5	38.2	
Number of hours per week worked in this hospital			0.13
Less than 20	35.5	35.4	
20–39	29.5	36.6	
40 or more	35.0	28.0	

^a^Mantel-Hanzel chi-square test; italics indicate *p*<0.05

^b^Other categories included registered nurses, licensed vocational nurses, licensed practical nurses, and surgical technicians

**Table 3:** Baseline characteristics of sampled hospitals in campaign and non-campaign states

	Campaign states (*n* = 5)	Non-campaign states (*n* = 5)	
Number of hospitals	*n* = 78	*n* = 95	*p*
Respondents per hospital (mean, range)	2.33 (1, 6)	2.45 (1, 6)	
	Mean (S.D.)		
Medicare hip/knee replacement procedure volume (2009)	250.1 (130.7)	270.4 (188.9)	0.42
	*N* (%)		
Ownership			0.66
Nonprofit	67 (85.7)	76 (80.0)	
For profit	6 (7.8)	11 (11.6)	
Public	5 (6.5)	8 (8.4)	
Part of hospital system/chain	55 (71.4)	68 (71.6)	0.98
Teaching status			
Member of Council of Teaching Hospitals (COTH)	6 (7.8)	14 (14.7)	0.23
Residency program	26 (33.8)	44 (46.3)	0.12
Located outside an MSA	16 (20.8)	12 (12.6)	0.21
	Mean (S.D.)		
Number of beds	270.5 (168.5)	280.5 (185.1)	0.72
Hospital quality and safety culture^a^			
Management support for patient safety	77.4 (24.3)	78.1 (24.5)	0.85
Teamwork across units	67.3 (27.0)	68.6 (26.5)	0.76
Handoffs and transitions	54.4 (31.2)	55.2 (32.4)	0.86

^a^Questions adapted from prior surveys of quality and patient safety culture [24]

Table 4 presents the main results after adjustment. In the campaign states, adherence to the three new evidence-based practices included in Project JOINTS increased and was statistically significant. Comparing the adherence change in campaign states to the adherence change in non-campaign states (difference-in-difference), the increase in adherence to the three new evidence-based practices ranged between 1.9 and 15.9 percentage points. Only the increase in adherence to the first of the three new practices (nasal screening and decolonization prior to surgery captured by three component measures) was statistically significant (*p* < 0.05). In the non-campaign states, none of the changes in adherence was statistically significant. By chance, baseline adherence was higher in non-campaign states than in campaign states on four of the five measures of the new bundle components. Baseline adherence to the measures previously featured in the SCIP program (timely receipt of prophylactic antibiotics, intravenous vancomycin for patients with MRSA, and appropriate hair removal) exceeded 94% in both campaign and non-campaign states.

**Table 4:** Change in reported use of evidence-based orthopedic surgery infection control practices in campaign and non-campaign states before and after the Project JOINTS campaign (italics indicate *p*<0.05 for the comparison)

	Campaign states			Non-campaign states			Campaign vs. non-campaign states (difference-in-difference)	
	Pre-intervention *n* = 77	Post-intervention *n* = 74		Pre-intervention *n* = 94	Post-intervention *n* = 91			
	% (S.D.)		*p*	% (S.D.)		*p*	Percentage point change	*p*
New evidence-based infection control practices								
1. Screening and decolonization								
1.a. Pre-operative nasal screening for *Staphylococcus aureus* carriage	*49.6 (4.5)*	*60.2 (4.4)*	*<0.0001*	59.0 (4.3)	60.7 (4.2)	0.558	*8.8*	*0.022*
1.b. Intranasal mupirocin prescribed for methicillin- resistant *S. aureus* (MRSA) carriers	*49.7 (4.3)*	*65.5 (4.2)*	*<0.0001*	71.9 (4.3)	78.0 (4.0)	0.090	*9.7*	*0.042*
1.c. Intranasal mupirocin prescribed for methicillin-sensitive *S. aureus* (MSSA) carriers	*37.0 (4.7)*	*55.0 (4.4)*	*<0.0001*	52.4 (4.8)	54.5 (4.4)	0.642	*15.9*	*0.008*
2. Chlorhexidine bathing 3 or more times preoperatively	*31.1 (4.1)*	*39.7 (3.8)*	*0.024*	34.1 (4.3)	36.2 (3.9)	0.624	6.5	0.263
3. Alcohol-containing antiseptic used to prepare skin in operating room	*96.9 (1.2)*	*99.0 (1.1)*	*0.019*	96.4 (1.2)	96.5 (1.1)	0.863	1.9	0.150
All-or-none composite of the new evidence-based infection control practices	*19.6 (3.8)*	*37.9 (5.1)*	*0.0007*	29.1 (4.9)	24.5 (4.3)	0.420	*23.0*	*0.004*
Previously promoted infection control practices from the Surgical Care Improvement Program (SCIP)								
4. Peri-operative intravenous antibiotics								
4.a. Timely receipt of prophylactic intravenous antibiotics	*98.7 (0.6)*	*99.7 (0.6)*	*0.027*	99.6 (0.6)	98.9 (0.6)	0.230	*1.6*	*0.017*
4.b. Intravenous vancomycin for MRSA	94.8 (1.1)	95.3 (1.1)	0.683	98.0 (1.2)	96.3 (1.2)	0.214	2.2	0.229
5. Appropriate hair removal techniques	94.2 (1.8)	94.4 (1.6)	0.901	94.8 (1.9)	93.6 (1.6)	0.575	1.4	0.615

On the all-or-none composite measure, the reported hospital adherence to all measures of the three new evidence-based bundle components increased from 19.6 to 37.9% in the campaign states, but declined slightly in the comparison states, yielding a relative increase (difference-in-difference) of 23 percentage points (*p* = 0.004).

## Discussion

In a cluster-randomized trial of a 6-month IHI quality improvement campaign, we found large increases in adherence to evidence-based practices that can prevent post-operative infection after hip and knee arthroplasty. Campaign states improved to a greater extent than non-campaign states. On measures of the practice of nasal screening and decolonization, the improvements were statistically significant in the difference-in-difference analysis (*p* < 0.05). On a pre-specified composite of reliable adoption of the three new evidence-based practice bundles, we found a statistically significant 23-percentage-point increase in campaign states relative to non-campaign states. These findings suggest that the substantial increased use of the three new evidence-based SSI prevention practices included in the bundle was attributable to the six-month campaign.

Over the past two decades, considerable effort has been devoted to developing effective methods for rapidly increasing the use of newly emerging evidence-based clinical practices and spreading their use across large numbers of organizations [28]. Most studies have focused on QI collaboratives and national initiatives that measure and report performance. These have shown effectiveness in some non-randomized studies (such as a statewide collaborative to reduce central-line associated bloodstream infections and the national D2B Alliance) [29, 30]. Yet the value of the collaborative approach remains unclear. Systematic reviews of QI collaboratives suggest that they are effective for some clinical situations and some settings but may produce modest or no improvement in others [3, 31]. Collaboratives can be resource intensive, usually involving face-to-face meetings of participants, up to 18-month implementation periods, and significant staff effort devoted by facilities that are under significant pressure to reduce costs. Recent efforts to reduce the duration of projects and engage participants virtually may address these challenges, but the effectiveness of “reduced effort” collaboratives is unclear.

The QI campaign is distinct from a collaborative in several ways. Like a political campaign, an improvement campaign orchestrates a carefully designed multi-faceted communications strategy, including tailored customer-specific messaging (e.g., for clinicians, patients, and administrators) and deploys diverse modes of delivery to engage relevant audiences [32]. A QI campaign is similar to other QI initiatives with a deliberate focus on a pre-specified set of evidence-based practices, but in addition, it leverages both professional and informal social networks within and across organizations to inform, motivate, and support the adoption and use of these practices. A campaign can take advantage of an established and potentially reusable dissemination structure (in this case, the Rapid Spread Network developed during IHI’s 100,000 Lives Campaign) to achieve timely dissemination of information and outreach to statewide organizations, hospital leaders, professional staff, and patients. It offers access to experts with clinical and QI knowledge, as well as organizations that have achieved superior performance and can inspire and mentor other organizations. It facilitates peer support and shared learning to achieve practice change.

QI campaigns do not require in-person meetings or require participants to collect and report extensive data. Anecdotally, campaigns seem best for spreading relatively well-defined, reasonably circumscribed clinical practices that are easy to message and to implement. Campaigns may be more effective among peer organizations that communicate actively with one another or among professions with a more cohesive identity and more tightly connected social networks. Local organizations that participate in communities of learning may be more receptive to campaigns. Until now, campaigns have been coordinated and driven by a central organization, but as social media and supporting technology platforms continue to evolve, the central organization may increasingly rely on a network of interactive, relatively independent learning communities in which innovative approaches to implementing evidence-based practices can be communicated and spread rapidly.

Few randomized, controlled evaluations of the QI campaign approach have been carried out. In the U.S., National campaigns, such as the D2B Alliance and the Partnership for Patients, made the intervention available to all potential participants, eliminating the option of control groups. Without control groups, observed improvements due to other factors may be erroneously attributed to the intervention [33]. In this study, we limited the campaign to intervention states by limiting publicity outside of the intervention states, using password-protected websites, and deferring participation requests from other states until after the study, when the campaign was released nationally. The lack of improvement in matched control states suggests that this cluster-randomized evaluation strategy was successful.

Our study has limitations. First, it is possible, though unlikely, that other national initiatives might have influenced practice. For example, the Partnership for Patients—a nationwide federal initiative that included efforts to reduce surgical site infection—was announced during Project JOINTS. However, we did not see improvement in the adoption of the evidence-based practices in our control states that were also exposed to the Partnership. Also, in our previously published qualitative research study, hospital leaders in the intervention states did not identify other initiatives with impact during the intervention period [13]. Second, in a cluster-randomized design residual imbalances could lead to differences between intervention and control states. To address this, we adjusted for respondent and organizational factors that differed between the intervention and control states using doubly robust multivariable methods. Third, we relied on staff reports of the use of evidence-based practices, which may have introduced various forms of reporting bias. The study design addressed this in several ways. We surveyed staff in both intervention and control states before and after the intervention, so if reporting bias were an issue, it would have to have differed in the campaign and non-campaign states. The survey avoided direct reference to the campaign. Our results suggest that staff differentiated among the bundle components we studied, reporting high rates of established infection control practices (i.e., peri-operative antibiotics) and variable use of the three newer evidence-based practices. Data on hospital-specific SSI rates would have been useful to corroborate our results, and those data were not available to us. Others have reported that use of the practices Project JOINTS targeted were associated with reduced infection rates [34].

Our results demonstrate the utility of cluster-randomized trial designs in assessing the impact of major improvement initiatives. This evaluation method including comparison groups and baseline measurements may be especially important as the Center for Medicare and Medicaid Innovation (CMMI) is introducing variety of innovative quality improvement initiatives in a short timeframe throughout the U.S. Baseline measures are critical because many measures may demonstrate variable or very high rates of adherence before intervention. Further improvement may not be possible under such circumstances. Finally, our study suggests that evaluation need not seriously delay implementation of effective interventions. The Project JOINTS campaign was opened to all states shortly after data collection was completed.

## Conclusions

In summary, in a cluster-randomized trial, we found that a quality improvement campaign increased the use of evidence-based practices that evidence demonstrates can reduce surgical site infections in hip and knee arthroplasty. We conclude that a carefully crafted campaign can accelerate the spread of evidence-based practices, scaling results from clinical trials and promising local initiative to larger regions and states. Application of the campaign method may be especially useful when newly emerging evidence suggests changes to current clinical care should be adopted rapidly as best practice. Given the substantial number of new evidence-based practices emerging each year, the campaign may be an efficient alternative or adjunct to other improvement scale-up methods.

## Additional file

Additional file 1: Simplified logic model for Project JOINTS Evaluation. (DOCX 53 kb)

## References

[1] Davis DA, Taylor-Vaisey A (1997). Translating guidelines into practice. A systematic review of theoretic concepts, practical experience and research evidence in the adoption of clinical practice guidelines. CMAJ.

[2] Schouten LM, Hulscher ME, van Everdingen JJ (2008). Evidence for the impact of quality improvement collaboratives: systematic review. BMJ.

[3] Nadeem E, Olin SS, Hill LC (2013). Understanding the components of quality improvement collaboratives: a systematic literature review. Milbank Q.

[4] Hulscher ME, Schouten LM, Grol RP (2013). Determinants of success of quality improvement collaboratives: what does the literature show?. BMJ Qual Saf.

[5] Berwick DM, Calkins DR, McCannon CJ (2006). The 100 000 lives campaign. JAMA.

[6] McCannon CJ, Hackbarth AD, Griffin FA (2007). Miles to go: an introduction to the 5 Million Lives Campaign. Jt Comm J Qual Patient Saf.

[7] Improvement IfH. How-to guide: running a successful campaign in your hospital. Secondary how-to guide: running a successful campaign in your hospital 2015. http://www.ihi.org/resources/Pages/Tools/HowtoGuideRunningSuccessfulCampaign.aspx. Aceessed 10 Apr 2017.

[8] McCannon CJ, Schall MW, Calkins DR (2006). Quality improvement: saving 100 000 lives in US hospitals. BMJ.

[9] McCannon C, Perla R (2009). Learning networks for sustainable, large-scale improvement. Jt Comm J Qual Patient Saf.

[10] Wachter RM, Pronovost PJ (2006). The 100,000 lives campaign: a scientific and policy review. Jt Comm J Qual Patient Saf.

[11] Schwamm LH, Fonarow GC, Reeves MJ (2009). Get With the Guidelines-Stroke is associated with sustained improvement in care for patients hospitalized with acute stroke or transient ischemic attack. Circulation.

[12] Nallamothu BK, Krumholz HM, Peterson ED (2009). Door-to-balloon times in hospitals within the get-with-the-guidelines registry after initiation of the Door-to-Balloon (D2B) Alliance. Am J Cardiol.

[13] Khodyakov D, Ridgely MS, Huang C (2015). Project JOINTS: what factors affect bundle adoption in a voluntary quality improvement campaign?. BMJ Qual Saf.

[14] Resar R, Pronovost P, Haraden C (2005). Using a bundle approach to improve ventilator care processes and reduce ventilator-associated pneumonia. Jt Comm J Qual Patient Saf.

[15] Bode LG, Kluytmans JA, Wertheim HF (2010). Preventing surgical-site infections in nasal carriers of Staphylococcus aureus. N Engl J Med.

[16] Kim DH, Spencer M, Davidson SM (2010). Institutional prescreening for detection and eradication of methicillin-resistant Staphylococcus aureus in patients undergoing elective orthopaedic surgery. J Bone Joint Surg.

[17] Paulson DS (1993). Efficacy evaluation of a 4% chlorhexidine gluconate as a full-body shower wash. Am J Infect Control.

[18] Byrne D, Napier A, Phillips G (1991). Effects of whole body disinfection on skin flora in patients undergoing elective surgery. J Hosp Infect.

[19] Kaiser A, Kernodle D, Barg N (1988). Influence of preoperative showers on staphylococcal skin colonization: a comparative trial of antiseptic skin cleansers. Ann Thorac Surg.

[20] Darouiche RO, Wall MJ, Itani KM (2010). Chlorhexidine–alcohol versus povidone–iodine for surgical-site antisepsis. N Engl J Med.

[21] Swenson BR, Hedrick TL, Metzger R (2009). Effects of preoperative skin preparation on postoperative wound infection rates: a prospective study of 3 skin preparation protocols. Infect Control Hosp Epidemiol.

[22] Langley G, Nolan T, Nolan K (1996). The improvement guide: a practical guide to enhancing organizational performance.

[23] Institute for Healthcare Improvement (2012). How-to guide: prevent surgical site infections.

[24] Singer S, Meterko M, Baker L (2007). Workforce perceptions of hospital safety culture: development and validation of the patient safety climate in healthcare organizations survey. Health Serv Res.

[25] Neugebauer R, van der Laan M (2005). Why prefer double robust estimators in causal inference?. J Stat Plan Inference.

[26] Funk MJ, Westreich D, Wiesen C (2011). Doubly robust estimation of causal effects. Am J Epidemiol.

[27] McCaffrey DF, Griffin BA, Almirall D (2013). A tutorial on propensity score estimation for multiple treatments using generalized boosted models. Stat Med.

[28] Bradley EH, Herrin J, Wang Y (2006). Strategies for reducing the door-to-balloon time in acute myocardial infarction. N Engl J Med.

[29] Dixon-Woods M, Bosk CL, Aveling EL (2011). Explaining Michigan: developing an ex post theory of a quality improvement program. Milbank Q.

[30] Bradley EH, Nallamothu BK, Herrin J (2009). National efforts to improve door-to-balloon time results from the Door-to-Balloon Alliance. J Am Coll Cardiol.

[31] Power M, Tyrrell PJ, Rudd AG (2014). Did a quality improvement collaborative make stroke care better? A cluster randomized trial. Implement Sci.

[32] McCannon CJ, Schall MW, Calkins DR (2006). Saving 100,000 lives in US hospitals. BMJ.

[33] Soumerai SB, McLaughlin TJ, Gurwitz JH (1998). Effect of local medical opinion leaders on quality of care for acute myocardial infarction: a randomized controlled trial [see comments]. JAMA.

[34] Schweizer ML, Chiang HY, Septimus E (2015). Association of a bundled intervention with surgical site infections among patients undergoing cardiac, hip, or knee surgery. JAMA.

