# Erratum to: Does a quality improvement campaign accelerate take-up of new evidence? A ten-state cluster-randomized controlled trial of the IHI’s Project JOINTS

**DOI:** 10.1186/s13012-017-0591-y

**Published:** 2017-05-10

**Authors:** Eric C. Schneider, Melony E. Sorbero, Ann Haas, M. Susan Ridgely, Dmitry Khodyakov, Claude M. Setodji, Gareth Parry, Susan S. Huang, Deborah S. Yokoe, Don Goldmann

**Affiliations:** 1 RAND Corporation, Santa Monica, CA USA; 2 Division of General Medicine and Primary Care, Brigham and Women’s Hospital, Boston, MA USA; 3 Department of Health Policy and Management, Harvard School of Public Health, Boston, MA USA; 4 Institute for Healthcare Improvement and Department of Pediatrics, Boston Children’s Hospital, Boston, MA USA; 5 Division of Infectious Diseases and Health Policy Research Institute, University of California Irvine School of Medicine, Irvine, CA USA; 6 Division of Infectious Diseases, Brigham and Women’s Hospital and Harvard Medical School, Boston, MA USA; 7 The Commonwealth Fund, One East 75th Street, New York, NY 10021 USA

## Erratum

Following the publication of the original article [1], it was brought to our attention that the title contained an error: “The Institute for Healthcare Improvement’s Project JOINTS” was incorrectly included as “the Institute for Health Improvement’s Project JOINTS”.

The title has been corrected to read: “Does a quality improvement campaign accelerate take-up of new evidence? A ten-state cluster-randomized controlled trial of the IHI’s Project JOINTS”.

The corrected title has been included in this erratum and updated in the original article.
