# Supplementary figures and images for: Does a quality improvement campaign accelerate take-up of new evidence? A ten-state cluster-randomized controlled trial of the Institute for Health Improvement’s Project JOINTS
Source: Implement Sci. 2017 Apr 17;12:51. doi: 10.1186/s13012-017-0579-7 (PMC5393011; doi:10.1186/s13012-017-0579-7)

Additional file 1. Simplified Logic Model for Project JOINTS Evaluation


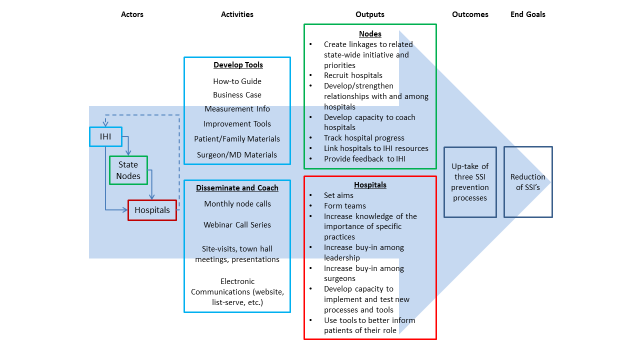

Supplement: Additional file 1: — Simplified logic model for Project JOINTS Evaluation. (DOCX 53 kb) [file 13012_2017_579_MOESM1_ESM.docx]
